# Supplementary material for: Concurrent Infection with SARS-CoV-2 and Pneumocystis jirovecii in Immunocompromised and Immunocompetent Individuals
Source: J Fungi (Basel). 2022 May 30;8(6):585. doi: 10.3390/jof8060585 (PMC9225243; doi:10.3390/jof8060585)
Supplement: Supplementary file 1 [file jof-08-00585-s001.zip › jof-1665517-supplementary.pdf]

Concurrent Infection with SARS-CoV-2 and Pneumocystis Jirovecii in immunocompromised and immunocompetent individuals

Francesca Gioia, Hanan Albasata and Seyed M Hosseini-Moghaddam

Data S1

Search strategies:

1. **Ovid MEDLINE(R) ALL**

((((((("COVID-19"[Mesh]) OR ("SARS-CoV-2"[Mesh])) OR ("Coronavirus"[Mesh])) OR ("covid-19")) OR (corona*)) OR ("sars-cov-2")) AND ((("Pneumocystis carinii"[Mesh]) OR ("Pneumonia, Pneumocystis"[Mesh])) OR (Pneumocystis))

2. **Embase**

('coronavirus disease 2019'/de OR 'severe acute respiratory syndrome coronavirus 2'/de OR 'coronavirus infection'/de OR 'covid 19':ab,ti OR 'corona*':ab, ti OR 'sars-cov-2':ab,ti) AND ('pneumocystosis'/exp OR pneumocystosis:ab,ti) AND [embase]/lim
